# Modification of the Human Amniotic Membrane Using Different Cross-Linking Agents as a Promising Tool for Regenerative Medicine

**DOI:** 10.3390/ma16206726

**Published:** 2023-10-17

**Authors:** Joanna Skopinska-Wisniewska, Marlena Michalak, Jakub Tworkiewicz, Dominik Tyloch, Marta Tuszynska, Anna Bajek

**Affiliations:** 1 Department of Chemistry of Biomaterials and Cosmetics, Nicolaus Copernicus University, Gagarina 7 Street, 87-100 Torun, Poland; 2 Department of Urology and Andrology, Ludwik Rydygier Collegium Medicum in Bydgoszcz, Nicolaus Copernicus University in Torun, Karlowicza 24 Street, 85-092 Bydgoszcz, Poland; 3 Chair of Urology and Andrology, Department of Tissue Engineering Ludwik Rydygier Collegium Medicum in Bydgoszcz, Nicolaus Copernicus University in Torun, Karlowicza 24 Street, 85-092 Bydgoszcz, Poland; marta.tuszynska96@gmail.com

**Keywords:** human amniotic membrane, cross-linking, aldehyde, EDC, NHS, squaric acid, regenerative medicine

## Abstract

Human amniotic membranes (hAMs) obtained during cesarean sections have proven to be clinically useful as an interesting biomaterial in a wide range of tissue engineering applications such as ocular surface reconstruction, burn treatments, chronic wounds, or bedsore ulcers. It presents antimicrobial properties, promotes epithelization, reduces inflammation and angiogenesis, contains growth factors, and constitutes the reservoir of stem cells. However, variability in hAM stiffness and its fast degradation offers an explanation for the poor clinical applications and reproducibility. In addition, the preparatory method of hAM for clinical use can affect its mechanical properties, and these differences can influence its application. As a directly applied biomaterial, the hAM should be available in a ready-to-use manner in clinical settings. In the present study, we performed an analysis to improve the mechanical properties of hAM by the addition of various reagents used as protein cross-linkers: EDC/NHS, PEG-dialdehyde, PEG-NHS, dialdehyde starch, and squaric acid. The effect of hAM modification using different cross-linking agents was determined via infrared spectroscopy, thermal analyses, mechanical properties analyses, enzymatic degradation, and cytotoxicity tests. The use of PEG-dialdehyde, PEG-NHS, dialdehyde starch, and squaric acid increases the mechanical strength and elongation at the breaking point of hAM, while the addition of EDC/NHS results in material stiffening and shrinkage. Also, the thermal stability and degradation resistance were evaluated, demonstrating higher values after cross-linking. Overall, these results suggest that modification of human amniotic membrane by various reagents used as protein cross-linkers may make it easier to use hAM in clinical applications, and the presented study is a step forward in the standardization of the hAM preparation method.

## 1. Introduction

Regenerative medicine is an interdisciplinary therapy dealing with principles and strategies of replacing, engineering or regenerating human cells, tissues, or organs to revive or establish their traditional, physiological functions. Within this field, three methodological approaches are observed: cell-based therapy, the usage of biomaterials or acellular scaffolds, and cell-seeded scaffolds. Decellularized scaffolds, due to their potential, have recently proven to have a capacity for medical use and organ repair and regeneration [1,2]. Increasing numbers of studies reveal that these biomaterials have the intrinsic ability to produce a positive regenerative microenvironment, and advance tissue-specific remodeling, they are also known as an inductive template for recellularization [3]. Mechanical properties and scaffold architecture, their biocompatibility, and biodegradability are considered the most important in scaffold designing processes. Human amniotic membrane (hAM) possesses many properties suited for the listed criteria, therefore it serves as a suitable material for use in regenerative medicine. Apart from supporting epithelization, it also affects inflammation, angiogenesis, or scarring. Its non-tumorigenic, antimicrobial, and antiviral properties are also observed, as well as several growth factors and some substances acting as proapoptotic agents. Moreover, the hAM cells have a pluripotent nature [3,4,5,6,7,8]. For those reasons, the amniotic membrane is attractive for tissue engineering and transplantation. It is used for ocular surface reconstruction, as a matrix for the construction of skin substitutes, especially for the treatment of burns, chronic wounds, bedsore ulcers, and skin defects [3,8,9,10,11]. It is also considered as a material for the reconstruction of urinary tract, bladder, vagina, and oral cavity. Moreover, hAM is tested as a matrix for ex vivo cultured limbal epithelial stem cells and as an autologous or allogeneic cell delivery systems [3,4,5]. Despite all of the advantages, there are some difficulties related to using amniotic membranes in regenerative medicine and clinical applications. Lack of a standardized protocol in hAM preparation and storage, heterogeneity, low mechanical strength of hAM, and a relatively fast degradation and susceptibility to rupturing during surgery are the most important problems [8,11,12,13,14,15]. To make it applicable for use in regenerative medicine, it will be necessary to improve its mechanical resistance without affecting other hAM properties. Many researchers have tried to improve the hAM degradation rate and mechanical properties by cross-linking processes. Various chemical reagents have been tested. However, the properties of the obtained materials are still not fully satisfactory. For example, glutaraldehyde was one of the most commonly used cross-linking agents in biomaterials chemistry, and it was also used for amniotic membrane modification. It reacts with amine groups in mild conditions and is incorporated into the protein network. The reaction is predictable, easy to control, and proceeds with high efficiency. However, the unreacted molecules can diffuse from the material to surrounding tissues and cause unwanted reactions [10,11,12,14,16,17]. For that reason, researchers are constantly looking for new safe, and effective cross-linking agents, which will improve the properties of amniotic membranes, thus making it a promising tool in tissue engineering and regenerative medicine.

Taking into account the main problems with hAM in the context of its application in clinical practice, in our study, we decided to investigate the influence of macromolecular cross-linking agents on the properties of human amniotic membranes. Dialdehyde starch (DA), polyethylene glycol-dialdehyde (PEG-A), and N-hydroxylsuccinimide (NHS) functionalized polyethylene glycol (PEG-NHS), which are considered a safer alternative for small-molecular dialdehyde and NHS reagents, were tested. Dialdehyde starch is a product of selective peroxidation of starch, in which glucose units are cleaved, and two aldehyde groups are formed. It is a biodegradable and effective cross-linker for amine groups. Also, PEG-A and PEG-NHS are amine-reactive compounds. They are formed as a result of modification of the hydroxyl end groups of the polyethylene glycol chain—the water-soluble, neutral, and biocompatible polymer widely used in pharmacy [18,19]. We also used squaric acid (SQ) (3,4-dihydroxy 3-cyclobutene 1,2-dione), which is a strong aromatic acid. Its rigid planar molecule contains two carboxyl groups and can create an ideally symmetrical dianion that willingly reacts with amine groups. Our previous experiments showed that squaric acid is an effective cross-linking agent for proteins, which suggests that it will also be useful for amniotic membrane modification [20]. The 1-ethyl-3-(3-dimethylaminopropyl)-1-carbodiimide hydrochloride/N-hydroxysuccinimide (EDC/NHS) as a typically used cross-linking system was also investigated.

## 2. Materials and Methods

### 2.1. Materials

Amniotic membranes were harvested from the human placenta during the cesarean section. All patients gave written informed consent and were informed about the procedure carried out according to the protocol of this study, which was approved by the Bioethics Committee of Nicolaus Copernicus University in Torun. The placenta was washed several times with BSS and antibiotics (50 μg/mL penicillin, 50 μg/mL streptomycin, 100 μg/mL neomycin, and 2.5 μg/mL amphotericin B). Next, the amniotic membrane was separated from the remaining parts of the placenta by tweezers. Then, the hAM was transported in a container, dipped in a medium consisting of Dulbecco Modified Eagle’s Medium (DMEM) and glycerol in the 1:1 ratio, and finally stored at −80 °C. The material was defrosted directly before cross-linking. It was cut into 15 × 10 cm pieces, put into plastic containers, and treated with 40 cm^3^ aqueous solutions of various cross-linking agents: 0.5% and 1% solutions of PEG-dialdehyde, MW 1000 g/mol, and 3000 g/mol (sample designation: 0.5-PEG-A1, 1-PEG-A1 and 0.5-PEG-A3, 1-PEG-A3); 0.5% and 1% solutions of O,O’-Bis[2-(N-Succinimidyl-succinylamino)ethyl]-polyethylene glycol, MW 3400 g/mol and 10,000 g/mol (sample designation: 0.5-PEG-NHS3, 1-PEG-NHS3 and 0.5-PEG-NHS10, 1-PEG-NHS10); 0.5% and 1% solutions of dialdehyde starch (sample designation: 0.5-DS, 1-DS; 0.5% and 1% solutions of squaric acid (sample designation: 0.5-SQ, 1-SQ); 0.025 mg and 0.050 mg of 1-ethyl-3-(3-dimethylaminopropyl)-1-carbodiimide hydrochloride per 1 mg of amniotic membrane. The EDC/NHS (N-hydroxysuccinimide) ratio was 5:1 (sample designation: I-EDC, II-EDC).

**Figure 1:**
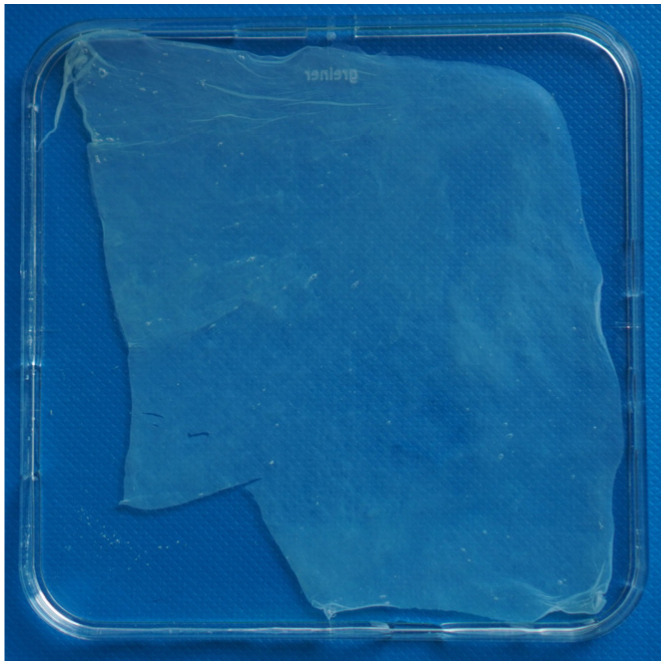
An example of the human amniotic membrane.

The hAM was immersed in appropriate solutions and left for 24 h, at 8 °C, under continuous stirring. After the cross-linking process, the materials were rinsed five times with deionized water to remove unreacted chemicals and then prepared for various analyses. An example picture of the human amniotic membrane is shown in Figure 1. All the cross-linked products did not differ from the one presented.

### 2.2. FTIR-ATR Spectroscopy

The small part of the various amniotic membranes was dried in the air. The FTIR-ATR spectra of samples were obtained with the Mattson Genesis II spectrophotometer (USA) equipped with an ATR tool, with a 4 cm^−1^ resolution, in the wavenumber range 4000–600 cm^−1^. 32 scans for each sample were collected. The FTIR spectra were compared using the program provided by the manufacturer.

### 2.3. Thermal Analysis—TG

The thermal properties of unmodified and cross-linked amniotic membranes were studied using a Simultaneous TGA-DTA Thermal Analysis TA Instruments, type SDT 2960. The samples were scanned at the temperature range from 20 °C to 600 °C and the heating rate of 10 °C/min in the atmosphere of nitrogen.

### 2.4. Mechanical Properties

The tensile strengths of hAM were determined using a Zwick&Roell Z 0.5 machine (Ulm, Germany) with an elongation speed 10 mm/min. The materials were cut into strips about 1 cm wide and 10 cm long and placed in deionized water until measurement. At least 5 samples of each kind of material were tested.

### 2.5. Enzymatic Degradation

The dried samples of the amniotic membrane material were weighed (*Wb*). Then, they were immersed in 1 cm^3^ of 0.1 M Tris-HCl buffer (pH 7.4) containing 50 mM CaCl_2_ and left at the temperature of 37 °C for 0.5 h. Next, 1 cm^3^ of 0.1 M Tris-HCl buffer (pH = 7.4) containing 50 U of bacterial enzyme–collagenase from Clostridium histolyticum (Sigma-Aldrich, Saint Louis, MO, USA) was added. After 1 h incubation at 37 °C, the samples were placed in 1 cm^3^ of 0.25 M EDTA, cooled in ice, rinsed 3 times with deionized water, frozen, lyophilized, and weighed (*Wa*). The degradation degree was calculated from the equation:

(1) $$Degradation\;degree\;\left(\%\right)=\left(Wb-Wa\right)/Wb\;*100$$

The value was an average of three measurements.

### 2.6. Biological Research

Biological research was conducted under sterile conditions at all times. The hAM samples were prepared following the protocol. Additionally, to improve the cell viability and prevent the toxic storage effect of hAM decomposition products due to the long storage period, which could have negatively affected the membrane itself, the untreated hAM was washed in PBS with antibiotics (penicillin, streptomycin, amphotericin B) for three days, changing the PBS solution every day. After the 24 h cross-linking in selected agents, the hAM was washed and stored for the extraction. Based on ISO 10993-12:2012 norm, the cross-linked amniotic membranes were cut out into 6 cm^2^ pieces of surface area and placed into the sterile Eppendorf tubes in 1 mL of DMEM. The extraction process was carried out at 37 °C for 24 h. Next, the extracts were taken out, diluted, and added to the mouse embryonic fibroblast cells (3T3 cell line) previously seeded onto the 96-well plates at a concentration of 5 × 10^4^ cells/well. The exposure of cells to the extracts was conducted for 24 h. The general MTT cytotoxicity assessment was performed. The absorbance measurement using a microplate reader at 570 nm and 655 nm wavelength was run.

## 3. Results

### 3.1. ATR-FTIR

Infrared spectroscopy is a useful tool for biological material analysis. It provides valuable information about the protein structure. The amniotic membrane is mainly composed of collagen; therefore, the FTIR analysis allowed us to discuss the influence of various cross-linking agents to hAM molecular organization.

**Figure 2:**
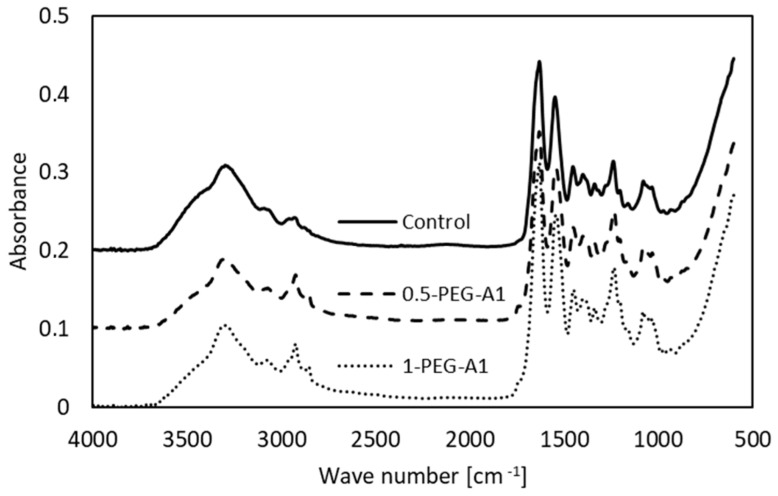
FTIR spectra of non-cross-linked amniotic membrane (Control) and modified by addition of 0.5% and 1% PEG-dialdehyde (MW 1000 g/mol).

The spectra of selected the tested amniotic membranes are shown in Figure 2.

The bands characteristic of collagen are observed. Amide A (~3300 cm^−1^) and Amide B (~3070 cm^−1^) are assigned to stretching vibrations of the N-H of the peptide bond, the frequency of which depends on the strength and symmetry of the hydrogen bond stabilizing the protein structure. These overlap with O-H and water molecules vibrations. In general, the position of the Amide A does not change but the Amide B is shifted (Table 1), and intensities of these bands decrease after amniotic membrane modification.

This suggests that the hydrogen bond architecture and water binding are rearranged due to cross-linking. Amide I (~1633 cm^−1^) arises from stretching vibrations of C=O and N-H, as well as bending vibrations of N-H of the peptide bonds. Its position depends on the nature of hydrogen bonds involving the peptide group, and is determined by backbone conformation. The maximum observed for hAM at 1631 cm^−1^ is attributed to the triple helical structure of native collagen. In some cases, the addition of a cross-linking agent (1-PEG-A3, 1-PEG-N3, 0.5-DS) changes the position of the band. This is accompanied by the alteration of the Amide II band (~1550 cm^−1^), which corresponds to N-H bending and C-N stretching vibrations. These irregular shifts of band position point that the collagen structure is altered. Moreover, the intensity of Amide I and Amide II is higher after modification, which shows that during the cross-linking process, new amide bonds are created. The addition of all the reagents causes an increase in the -CH_2_ band intensity, and two maxima instead of one are observed, which results from the polymeric structure of cross-linkers. We can also notice a new peak around 1740 cm^−1^, related to C=O of aldehydes, that arises after modification [4,16,21,22,23,24,25].

### 3.2. Thermal Stability

**Figure 3:**
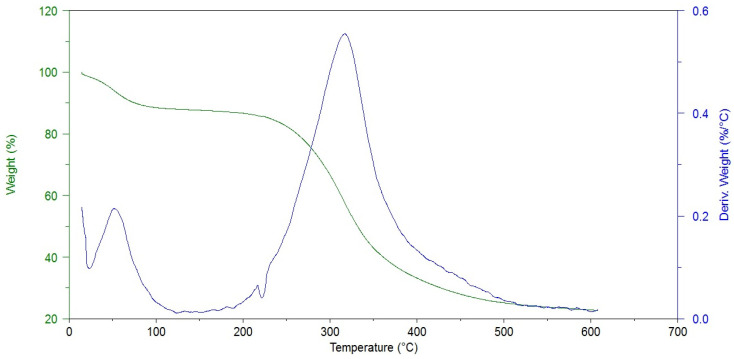
Thermogram of non-cross-linked amniotic membrane (Control).

The thermogravimetric analysis allowed us to determine the sample moisture content and the degradation temperature, which may be related to the presence of cross-linking bonds. As one can see in Figure 3, the thermal decomposition of hAM proceeds in two stages.

The first one is associated with the evaporation of water from the material. The modified amniotic membranes show higher dehydration temperatures, i.e., 33.6 °C for hAM and 42–54 °C for cross-linked materials. At the same time, the weight loss is lower after the cross-linking process (19.7% and 5.7–18.2%), Table 2.

This stays in agreement with the FTIR analysis results (decrease in Amide A intensity). The second stage is related to the thermal degradation of the protein structure and occurs in a wide temperature range of 200–500 °C. The maximum decomposition of the amniotic membrane was observed at 315.4 °C, which is consistent with literature data [24]. The temperature of the maximum weight loss increases after hAM modification and the mass losses are smaller in most cases. These prove that the protein structure was cross-linked by the addition of all kinds of reagents used.

### 3.3. Mechanical Properties

Despite excellent biological properties, the use of amniotic membranes in tissue engineering is limited due to unsatisfactory mechanical properties. They tear relatively easily and are difficult to manipulate during application because of their high flaccidity [26]. Therefore, an attempt was made to modify the mechanical properties of hAM by cross-linking with various agents. Since they are usually used in the moist environment of the wound, eye surface, or inside the body, the mechanical properties of hAM were tested for membranes in the hydrated state. For this reason, the membranes were cut into strips, placed in deionized water, removed immediately before the measurement, and mounted in the measurement holders without drying.

**Figure 4:**
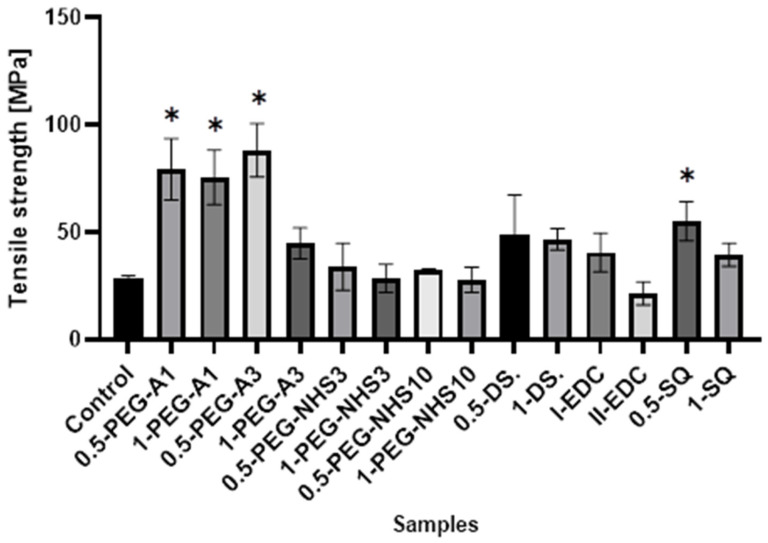
The values of tensile strength of amniotic membranes are cross-linked by the addition of PEG-dialdehyde, PEG-NHS, dialdehyde starch, EDC/NHS, and squaric acid. * Significant differences (*p* < 0.05) when compared to Control, performed via one-way analysis of variance (ANOVA) as the mean ± SD.

All the cross-linked materials except II-EDC show a tensile strength higher than the natural membrane (Figure 4), wherein hAM modified by PEG-dialdehyde and dialdehyde starch are the strongest. This shows that cross-linking with the aldehyde groups is more effective than using compounds with succinimide groups. Interestingly, the increase in the film strength is not proportional to the amount of a cross-linking agent. The materials treated with a higher amount of cross-linker are frequently weaker. Also, squaric acid, when used at a concentration of 0.5%, significantly improves the tensile strength of hAM. However, using a larger amount of SQ has a smaller impact on the strength of the material. A similar effect was reported in another paper regarding gelatin cross-linking by various reagents [27]. This may be because the amniotic membrane is a complex and compact material, and cross-linking occurs mainly on the surface of the material and also depends on the hAM’s protein composition.

**Figure 5:**
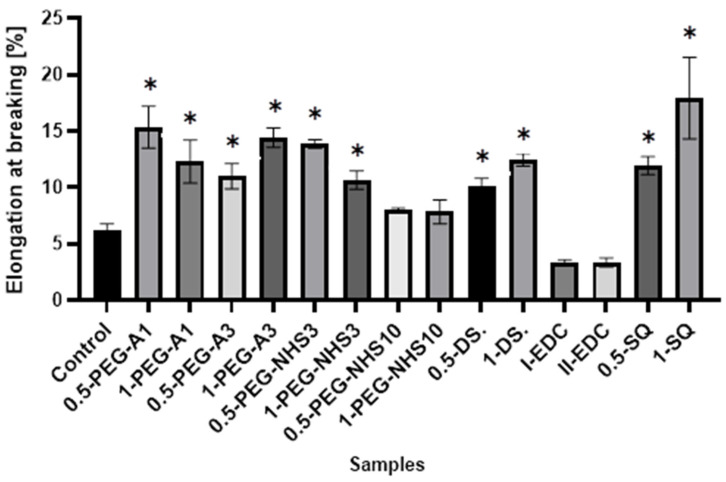
The values of elongation at the breaking point of amniotic membranes cross-linked by addition of PEG-dialdehyde, PEG-NHS, dialdehyde starch, EDC/NHS, and squaric acid. * Significant differences (*p* < 0.05) when compared to Control, performed via one-way analysis of variance (ANOVA) as the mean ± SD.

**Figure 6:**
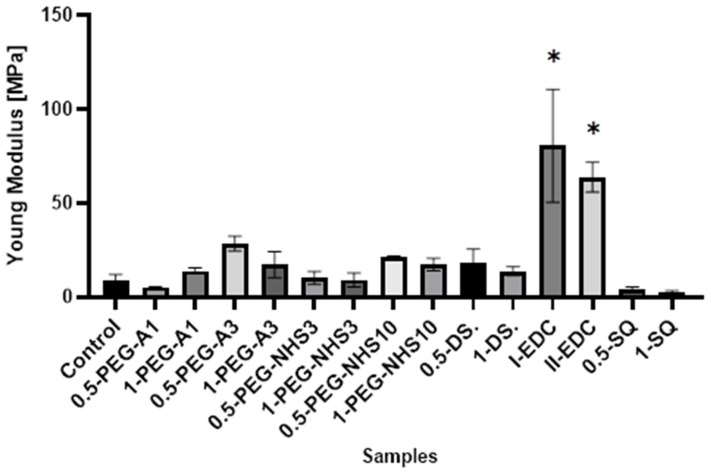
The values of Young’s modulus of amniotic membranes cross-linked by addition of PEG-dialdehyde, PEG-NHS, dialdehyde starch, EDC/NHS, and squaric acid. * Significant differences (*p* < 0.05) when compared to Control, performed via one-way analysis of variance (ANOVA) as the mean ± SD.

Interestingly, in most cases, the elongation at the breaking point increases after cross-linking (Figure 5). However, as in the case of tensile stress, there is no direct correlation between the amount of cross-linking agent and the value of a parameter. At the same time, a significant increase in Young’s modulus was noted for these materials (Figure 6). In the case of the other cross-linked hAMs, a very diverse impact of cross-linking on the material’s stiffness is observed. Typically, polymer crosslinking agents with higher molecular weights (PEG-A3, PEG-NHS10, DS) stiffen the membranes, increasing Young’s modulus value, while compounds with shorter chains do not cause this effect.

### 3.4. Degradation

As previously mentioned, collagen is one of the key components of the amniotic membrane. It is resistant to various enzymes and can degrade completely only with collagenase, which cuts the peptide bonds between a neutral amino acid and glycine. The enzymatic degradation of collagen materials depends on many various factors, e.g., the content of triple-helix in the protein structure, cross-linking degree, water content, and the availability of cleavage sites [12,16]. It occurs at physiological pH and 37 °C. The applied method is only a simple simulation of the degradation process, which is much more complex in vivo and gives only an overall look at the resistance of the material to enzymatic degradation.

**Figure 7:**
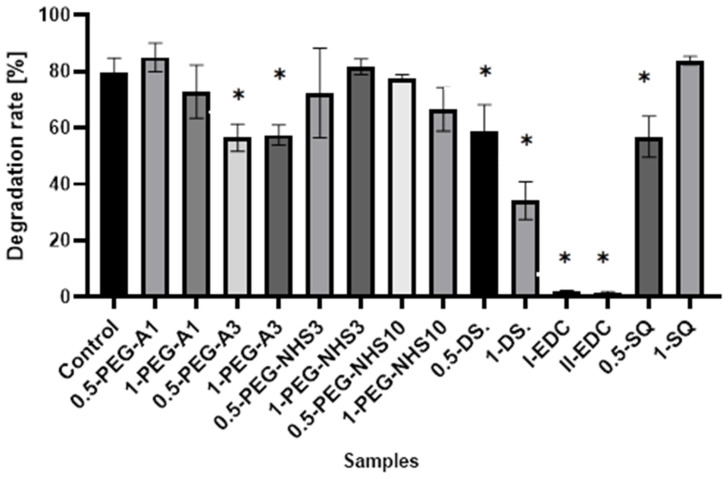
The susceptibility to enzymatic degradation of amniotic membranes cross-linked by addition of PEG-dialdehyde, PEG-NHS, dialdehyde starch, EDC/NHS, and squaric acid. * Significant differences (*p* < 0.05) when compared to Control, performed via one-way analysis of variance (ANOVA) as the mean ± SD.

The results presented in Figure 7 show that cross-linking of amniotic membranes affects their enzymatic degradation. The samples with the EDC/NHS addition are most resistant to degradation. Less than 2% of hAM is degraded by collagenase within 1 h, while 80% weight loss for natural hAM is observed. Cross-linking by PEG-dialdehyde MW 3000 g/mol, dialdehyde starch, and 0.5% SQ also decreases susceptibility to enzymatic degradation but to a lesser extent. The addition of other cross-linkers only slightly affects the amniotic membrane degradation resistance.

### 3.5. Biological Research

**Figure 8:**
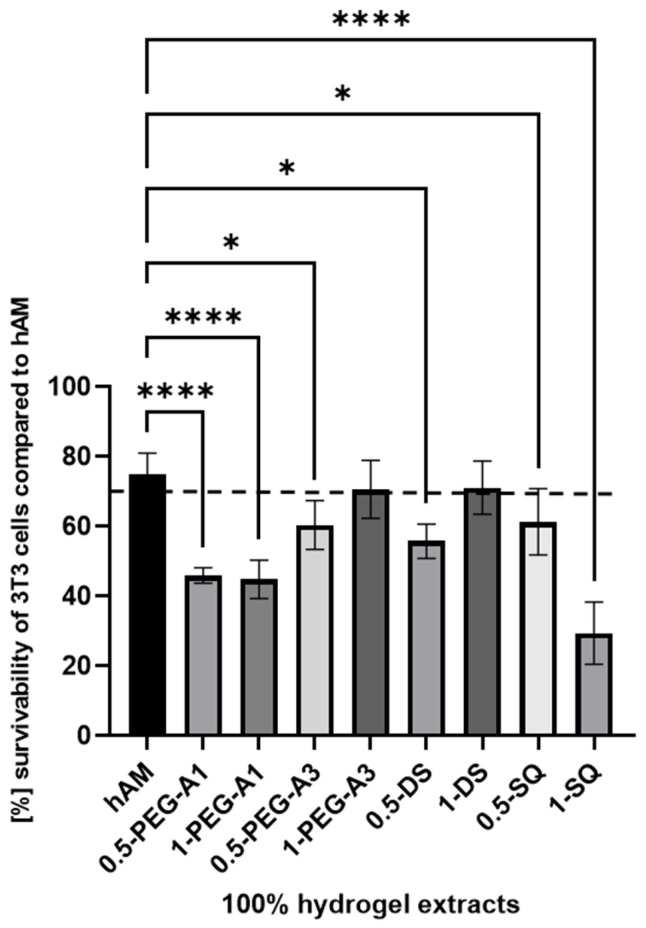
The survivability of 3T3 cells compared to hAM after 24 h of incubation with 100% extract concentration. The results by ANOVA are expressed as the mean ± SD, and * *p* < 0.02 **** *p* < 0.0001 or lower indicates a significant difference.

The cytotoxicity analysis, such as MTT assay, is performed to assess the cellular metabolic activity as an indicator of cell viability, proliferation, and cytotoxicity. It consists of extracting samples in DMEM according to ISO 10993-12 norm and then exposing the 3T3 cells to the extracts for a specified time. The obtained cytotoxicity results of extracts from the hMA are presented in Figure 8. The biological research was performed using only selected cross-linkers (0.5% and 1% concentration of PEG-A1, PEG-A3, DAS, and SQ), as they showed the most optimal and promising chemical parameters. The derived results were then compared to extracts obtained for untreated hAM.

The obtained data revealed that hAM alone, despite thorough washing during the preparation process, showed only slightly above 70% 3T3 cell viability. Data derived for the 1-PEG-A3 and 1-DAS reached norm-defined cell survivability. That confirms that hAM treated with PEG-dialdehyde MW 3000 g/mol, dialdehyde starch at 1% concentration, is improved. Other tested samples resulted as more toxic. Especially, SQ in a higher concentration is the most harmful for the cells, as it creates a more acidic, thus unfavorable, cell environment. Interestingly, the PEG-dialdehyde MW 1000 g/mol did not change between samples, which also resulted in the low viability of cells.

## 4. Discussion

The regenerative medicine is a commonly used name indicating the use of biotechnological applications to obtain the regeneration or repair of tissues and also the whole organs. Various methods have been developed up to now, and several scaffolds have been used. There are many reports in the literature on the biological properties of human amniotic membranes, such as low immunogenicity, anti-inflammatory properties, or the ability to promote cell differentiation. Many studies have been carried out using hAM as a substrate for the culture of various types of epithelial cells [4,28,29,30], as well as for the treatment of corneal damage [10,11,16,28,29,31], skin loss [32], in heart tissue regeneration [7], reconstructive urology [13,33], and as a wound dressing [17,34,35]. Besides the aforementioned properties of hAM, antifibrotic and antimicrobial ones are also beneficial for clinical applications. However, there are fewer reports on the physicochemical properties of these materials, which is crucial for clinical application. The mechanical properties, susceptibility to damage during surgery, and degradation resistance strongly depend on the cross-linking degree of the material. Moreover, several papers show that the material’s stiffness is significant for cell proliferation and differentiation, which seems to be important in cell-based therapies [28,34,36,37]. This parameter can also be modified by cross-linking the material. Therefore, in our research, we focused on determining the effectiveness of the human amniotic membrane cross-linking with the use of various small-molecular, as well as polymeric, reagents. Our study has shown that all the used reagents cause the formation of cross-links and change the internal architecture of the amniotic membrane. This is proved by the increase in the thermal degradation temperature of cross-linked materials, as well as the changes observed in the FTIR spectra. The increase in Amide I and Amide II intensity indicates the formation of new amide bonds as a result of reactions between aldehyde groups of DAS, PEG-A, and amino groups of hAM [18,19], and also mediated by EDC/NHS and PEG-NHS [4]. As a result, the mechanical strength of cross-linked human amniotic membranes is improved. The tensile strength of tested natural hAM is 28.5 ± 1.5 MPa, and the percentage elongation is 7.4 ± 1.4%. There are various data in the literature, from 1.6 MPa to more than 20 MPa [14,16,24,38,39]. These differences may be due to the fact that amniotic membranes are natural and diverse materials, and their properties are affected by various independent factors such as age, diet, and the health of the woman. Moreover, Conon et al. showed that the structure of the human amniotic membrane differs in various areas [40]. This also could affect the mechanical properties of the material. The tensile strength grows even up to 80 MPa after modification with PEG-A. Interestingly, elongation at the breaking point increases after cross-linking with most of the reagents used. As it is well known, aldehydes easily react with amino groups under mild conditions [14,19]. Hence, the high effectiveness of crosslinking using DAS and PEG-A is observed. The formation of cross-links using NHS-containing reagents is less effective, as evidenced by the lower strength of the materials. However, it should be remembered that macromolecular cross-linking agents were used, and the size of the particles limits the ability of cross-linking agents to penetrate into the deeper layers of the amniotic membrane. Therefore, material modification occurs mainly on the surface. It can also be supposed that long polymer chains limit the contact of the functional groups of the cross-linking agent with the amino groups on the hAM surface. As a result, PEG-NHS weighing 10,000 has the lowest impact on the material’s mechanical properties among the high-molecular reagents. We assume that using macromolecular compounds enables cross-linking but, at the same time, affects the material’s properties through the mere presence of long chains on the membrane surface and the occurrence of non-covalent intermolecular interactions. The changes in properties are quite irregular. A larger amount of polymer does not increase the film’s strength, but may sometimes improve elasticity. The cross-linking with EDC/NHS causes significant stiffening and shrinkage of the hAM. Carbodiimide is a zero-length cross-linker that induces the formation of numerous amide bonds between the amino and carboxyl groups in proteins. This causes stiffening of the hAM membrane and increases the fragility of the material, but at the same time, it significantly increases the resistance of hAM to degradation by collagenase. Interestingly, cross-linking with the use of squaric acid not only improves the strength and elasticity of hAM, but also makes it adhesive, and combining several layers of amniotic membranes is possible. Square acid is also very effective at forming cross-links. It is a low-molecular, aromatic reagent with a strongly acidic character. The presence of two carboxyl groups in an asymmetric molecule causes extensive delocalization of π electrons on all oxygen and carbon atoms and an evenly distributed negative charge. Thanks to this, it can react with amino groups but also interact with charged side groups of the protein. Hence, due to various mechanisms of interactions, the modification effects may be disproportionate to the amount of reagent used, and it is necessary to select the optimal amount of cross-linking agent for a specific system [20,27]. Similar imparts to the elasticity of the material but also reduction in tensile strength was observed by Ma et al. and Gnanasundaram et al. during hAM cross-linking using glutaraldehyde [8,24]. However, due to the acidic effect of the pure squaric acid solution, the treated amniotic membrane performed the lowest viability of 3T3 cells exposed to the 0.5-SQ and 1-SQ extract from hAM.

There are many reports about hAM’s healing properties. However, it seems that the storage method of hAM is crucial for its efficiency and application. The most popular preservation methods, like storage at −4 °C, −70 °C, or lyophilization, are impractical at specific clinical conditions and applications. Many studies have shown reduced cell viability after hAM storage [34,35,41,42,43,44,45,46,47]. This may also be the cause of low cytotoxicity results obtained for our nontreated hAM and, as a consequence, also for cross-linked materials. Our previous works showed that the cross-linking factors used in this research support cells’ attachment and growth when used for collagen, elastin, or gelatin cross-linking, and they can be applied for modification of biomaterials for tissue regeneration and used as a safe alternative for popular cross-linking agents [18,19,20,27]. We expect a similar effect in the case of hAM modification, but further studies should be conducted considering how the amniotic membrane is stored.

## 5. Conclusions

In many clinical applications, there is a huge need for the stabilization of biomaterials such as hAM before transplantation. Cross-linking is one of the methods used for increasing the biomechanical strength of transplants.

The presented results show that cross-linking is an effective approach to amniotic membrane properties modification. The improvement in thermal stability, as well as changes observed on infrared spectra of the tested materials, prove the cross-linking process occurrence. The mechanical strength and elongation at the breaking point of hAM increase due to cross-linking by PEG-dialdehyde, PEG-NHS, dialdehyde starch, and squaric acid, while the use of EDC/NHS causes stiffening and shrinkage of the material. Interestingly, the 0.5% cross-linking agent addition gives better results than the 1% addition. However, these changes in mechanical properties make the amniotic membrane easier to manipulate during surgery. Degradation resistance is improved after hAM treatment with PEG-dialdehyde MW 3000 g/mol, dialdehyde starch, and 0.5% squaric acid; but the highest resistance is definitely observed for hAM with EDC/NHS. Likewise, the cross-linking using 1-PEG-A3 and 1-DAS supports cell attachment and growth. In general, the use of aldehyde cross-linking agents (PEG-dialdehyde and dialdehyde starch) is more effective than applying NHS derivatives. Also, the use of 0.5% SQ gives very promising results. It improves the mechanical properties and degradation resistance of hAM, as well as increasing its adhesiveness. We believe that such modified hAM can serve as a potential biomaterial for regenerative medicine and clinical applications, and the presented study is a step forward standardization of the hAM preparation method.

## Figures and Tables

**Table 1:** The position of main bands in FTIR spectra of non-modified and cross-linked amniotic membrane.

Sample	Amide A [cm^−1^]	Amide B [cm^−1^]	=CH_2_ [cm^−1^]	C=O [cm^−1^]	Amide I [cm^−1^]	Amide II [cm^−1^]	Amide III [cm^−1^]
Control	3298	3092	2933	-	1631	1548	1239
0.5-PEG-A1	3301	3072	2925 2854	-	1632	1544	1236
1-PEG-A1	3300	3074	2925 2854	1743	1632	1542	1236
0.5-PEG-A3	3300	3074	2924 2854	-	1632	1548	1238
1-PEG-A3	3285	3062	2923 2853	1737	1647	1538	1234
0.5-PEG-NHS3	3301	3075	2924 2854	-	1631	1549	1239
1-PEG-NHS3	3284	3072	2923 2853	1741	1645	1540	1239
0.5-PEG-NHS10	3300	3074	2926 2854	-	1632	1538	1235
1-PEG-NHS10	3300	3074	2924 2854	1738	1631	1549	1239
0.5-DS	3282	3063	2922 2852	1737	1651	1538	1235
1-DS	3300	3074	2926 2854	1739	1632	1548	1238
I-EDC	3298	3089	2924 2854	1745	1632	1547	1238
II-EDC	3287	3073	2925 2854	1743	1632	1546	1238
0.5-SQ	3283	3063	2922 2853	1742	1655	1538	1234
1-SQ	3285	3075	2923 2853	1742	1645	1538	1236

**Table 2:** Thermal parameters for the thermal decomposition of non-modified and cross-linked amniotic membrane.

Sample	T_1 max_ [°C]	Δm_1_ [%]	T_2 max_ [°C]	Δm_2_ [%]
Control	33.6	19.70	315.4	75.91
0.5-PEG-A1	49.9	5.71	321.1	47.50
1-PEG-A1	51.7	13.17	316.4	62.64
0.5-PEG-A3	42.5	11.48	317.5	73.20
1-PEG-A3	49.0	11.69	319.6	62.33
0.5-PEG-NHS3	51.4	9.36	319.1	45.13
1-PEG-NHS3	49.7	8.66	319.9	59.34
0.5-PEG-NHS10	51.2	18.09	316.5	43.15
1-PEG-NHS10	49.7	15.29	317.8	75.38
0.5-DS	52.9	10.26	319.5	57.35
1-DS	50.0	10.49	317.1	56.85
I-EDC	53.9	18.24	318.8	65.25
II-EDC	49.7	14.91	320.3	85.08
0.5-SQ	51.7	13.83	318.5	77.46
1-SQ	51.9	11.65	316.8	63.85

## Data Availability

The data presented in this study are available on request from the corresponding author.
